# The acute management of trauma hemorrhage: a systematic review of randomized controlled trials

**DOI:** 10.1186/cc10096

**Published:** 2011-03-09

**Authors:** Nicola Curry, Sally Hopewell, Carolyn Dorée, Chris Hyde, Karim Brohi, Simon Stanworth

**Affiliations:** 1 NHS Blood and Transplant, Oxford Radcliffe Hospitals NHS Trust and University of Oxford, Headley Way, Oxford, OX3 9BQ, UK; 2 Systematic Review Initiative (SRI), NHS Blood and Transplant, John Radcliffe Hospital, Oxford, Headley Way, Oxford, OX3 9BQ, UK; 3 UK Cochrane Centre, 18-24 Middle Way, Summertown, Oxford, OX2 7LG, UK; 4 Peninsula Technology Assessment Group (PenTAG), Peninsula College of Medicine and Dentistry, University of Exeter, EX2 4SG, UK; 5 Trauma Sciences, Bart's and the London School of Medicine and Dentistry, Queen Mary University of London, London, E1 4NS, UK

## Abstract

**Introduction:**

Worldwide, trauma is a leading cause of death and disability. Haemorrhage is responsible for up to 40% of trauma deaths. Recent strategies to improve mortality rates have focused on optimal methods of early hemorrhage control and correction of coagulopathy. We undertook a systematic review of randomized controlled trials (RCT) which evaluated trauma patients with hemorrhagic shock within the first 24 hours of injury and appraised how the interventions affected three outcomes: bleeding and/or transfusion requirements; correction of trauma induced coagulopathy and mortality.

**Methods:**

Comprehensive searches were performed of MEDLINE, EMBASE, CENTRAL (*The Cochrane Library *Issue 7, 2010), Current Controlled Trials, ClinicalTrials.gov, the World Health Organization International Clinical Trials Registry Platform (ICTRP) and the National Health Service Blood and Transplant Systematic Review Initiative (NHSBT SRI) RCT Handsearch Database.

**Results:**

A total of 35 RCTs were identified which evaluated a wide range of clinical interventions in trauma hemorrhage. Many of the included studies were of low methodological quality and participant numbers were small. Bleeding outcomes were reported in 32 studies; 7 reported significantly reduced transfusion use following a variety of clinical interventions, but this was not accompanied by improved survival. Minimal information was found on traumatic coagulopathy across the identified RCTs. Overall survival was improved in only three RCTs: two small studies and a large study evaluating the use of tranexamic acid.

**Conclusions:**

Despite 35 RCTs there has been little improvement in outcomes over the last few decades. No clear correlation has been demonstrated between transfusion requirements and mortality. The global trauma community should consider a coordinated and strategic approach to conduct well designed studies with pragmatic endpoints.

## Introduction

Trauma is one of the world's leading causes of death and disability. Around 40% of deaths are due to bleeding or its consequences, establishing hemorrhage as the most common cause of preventable death in this clinical group [1-3]. The relationship between trauma hemorrhage and poor outcomes has been well recognized for over 30 years [4], and applies globally [5,6], in both civilian and military settings [7]. However, outcomes from severe hemorrhage remain poor, with mortality rates approaching 50% for patients who require massive blood transfusion or who develop a significant coagulopathy [8,9]. Management of trauma hemorrhage depends on a multifactorial approach of timely surgical intervention, fluid resuscitation and blood transfusion therapy [10].

Advances have taken place in our understanding of the pathophysiology of trauma induced coagulopathy [11,12], in the availability of rapid diagnostic modalities [13], and the introduction of hemostatic resuscitation strategies [14]. Conversely, evidence reviews have shown that some accepted therapies such as blood or plasma transfusion may be ineffective or associated with worse outcomes [15,16].

Existing reviews have focused on individual interventions, such as transfusion ratios [16-19], blood substitutes [20], or pharmaceutical agents [21,22]. Our objective was to conduct a systematic review of the wider trial literature for all randomized controlled trials (RCTs) relevant to the early management of trauma patients with bleeding. We specifically aimed to appraise the methodology of the trials and to assess a broad range of outcomes focusing on bleeding and transfusion requirements, correction of coagulopathy and mortality.

## Materials and methods

### Search strategy

We followed a study specific protocol for this systematic review. All RCTs relating to early management of hemorrhage, transfusion or traumatic coagulopathy in severely injured patients of any age were considered for inclusion. No language restrictions were set. MeSH index and free text terms combined with RCT filters were used to search MEDLINE (1950 to July 2010), EMBASE (1980 to July 2010), and CENTRAL (*The Cochrane Library *Issue 7, 2010). We searched the ongoing trial registers: Current Controlled Trials, ClinicalTrials.gov and the World Health Organization International Clinical Trials Registry Platform (ICTRP). The National Health Service Blood and Transplant Systematic Review Initiative (NHSBT SRI) RCT Handsearch Database (1980 to July 2010) and the Cochrane Injuries Group Specialist Register were searched, and the reference lists of the identified RCTs and relevant narrative reviews were checked for additional trials. Papers not published in English were translated. Full details of the search are presented in Additional file 1.

### Selection criteria

Citations and abstracts identified by the searches were screened for relevance by one reviewer. Full publications of accepted studies were assessed by two reviewers working independently against the inclusion/exclusion criteria. The criteria for inclusion of full reports were: at least 75% of the subjects were trauma patients with bleeding or hemorrhagic shock; interventions were applied within 24 hours of injury; the RCTs compared treatment and placebo or alternative treatments; outcomes reported included bleeding, blood loss, coagulopathy, or transfusion requirements; and allocation of the groups was by formal randomization or a quasi-random method. Data were recorded on mortality and morbidity including multi-organ failure (MOF), acute respiratory distress syndrome (ARDS) and infection. Trials assessing isolated traumatic brain injury or burns were excluded.

### Data abstraction and quality assessment

Data were abstracted onto study specific forms by one reviewer and verified by a second reviewer. This included: country of origin, clinical setting, study population, trial structure, study quality, nature and duration of intervention and control groups, outcomes assessed and conclusions reported. Disagreements were resolved by consensus. Assessment of the methodological quality of the eligible RCTs was undertaken. We assessed the generation of random sequence, concealment of allocation, blinding of allocation and incomplete outcome data [23].

### Analysis

We performed a descriptive analysis as it was not possible to undertake a meta-analysis due to the heterogeneity of the interventions. The RCTs were grouped into four clinical areas: blood and blood saving strategies; mechanical and surgical management; use of intravenous fluids for resuscitation; and pharmaceutical agents.

## Results

**Figure 1:**
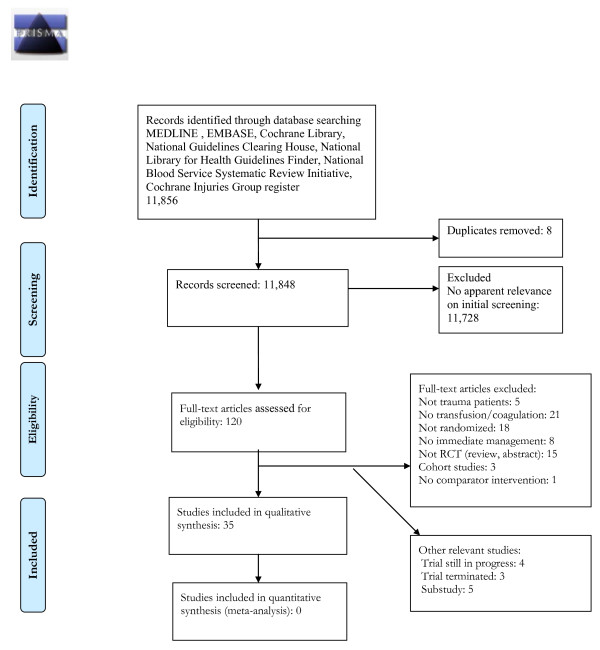
**PRISMA Flow Diagram for immediate bleeding management in trauma patients**.

The search strategy identified 11,856 citations. A total of 120 citations were relevant and reviewed at full text. After exclusions (Figure 1) [24], 35 completed RCTs were eligible for analysis (Additional file 2) [25-63]. Four trials are ongoing [64-67] (Table 1) and three have been terminated [68-70] (Table 2). Trials ranged in size from 32 to 20,211 participants and the majority (*n *= 23) were single centre studies. Thirty-four trials were of parallel group design and one a crossover trial [49]. Nine studies examined a pre-hospital intervention [32,34,41-44,47-49], one study used an intervention in both pre-hospital and hospital settings [31] and the remaining interventions were administered in-hospital [25,26,29,30,33,35-40,45,46,50-57,61-63].

**Table 1:** On-going studies

On-going study	Clinical group of trauma patients	Intervention details	Comparator details	Primary endpoint	Target number to be recruited	Expected end date
**CRISTAL: Colloids versus crystalloids for resuscitation of critically ill patients**	ITU patients, fluid resuscitation	Colloids	Crystalloids	28 day mortality	3,010	March 2011
**High versus low MAP for trauma patients undergoing surgery**	Adults, SBP < 90 mmHg, requiring laparotomy or thoracotomy,	Target minimum mean arterial BP 50 mmHg	Target minimum mean arterial BP 65 mmHg	30 day survival	271	July 2011
**FIRST: Colloids versus crystalloids for resuscitation of trauma patients**	Adults, requiring ≥3 litres of fluid	HES 130/0.4 in saline (Voluven)	0.9% saline	Fluid volumes over first 24 hours	140	December 2009
**Formula-driven vs. laboratory-guided transfusion in bleeding trauma patients: a feasibility study**	Adults, requiring four units of RBC in two hours and ongoing blood loss	FFP:RBC:platelets ratio of 1:1:1 - formula	Standard of care	Protocol compliance at 12 hours	70	October 2011

FFP, fresh frozen plasma; GCS, Glasgow coma score; ITU, intensive care unit; MAP, mean arterial pressure; RBC, red blood cell; SBP, systolic blood pressure.

**Table 2:** Terminated studies

Study	Clinical group of trauma patients	Intervention details	Comparator details	Primary endpoint	Completion/Termination date
**Warming techniques for treatment of hypothermia in polytrauma**	Adults, polytrauma, GCS > 9, ISS > 16 and ASCOT score = 2 to 50%	Endovascular catheter + forced air warming	Forced air warming	Morbidity during length of stay	Suspended July 2010. Insufficient numbers of patients recruited
**Hypertonic fluids for resuscitation of hypovolemic shock**	Adults, prehospital SBP ≤ 70, or prehospital SBP 71-90 and HR ≥108	Arm A: 7.5% hypertonic saline/6% Dextran-70 Arm B: 7.5% hypertonic saline **three arm trial**	Arm C: 0.9% normal saline	28-day survival	Terminated August 2009 - no difference in 28-day survival (futility). Analysis reported earlier but not higher mortality with hypertonic saline arms.
**Low dose vasopressin versus placebo in Traumatic Shock Resuscitation**	Adults, SBP < 90 mmHg	Bolus vasopressin 4 U, then continuous infusion 2.4 U/hour for five hours	Normal saline	To develop new resuscitation regimens	Terminated April 2009 - poor accrual rate

ASCOT, a severity characterization of trauma score; rFVIIa, recombinant activated factor VII; SBP, systolic blood pressure.

The majority of trials (*n *= 31) recruited trauma patients exclusively, but four studies included non-trauma patients comprising between 4 and 25% of participants [25,32,45,46], totalling 81 patients. All 35 studies included civilian patients only. Six trials only recruited participants with penetrating injuries [29,34,35,41,48,57] and one only blunt injury [57]. The 22 studies that included both types of injury had a mean penetrating injury rate of 37% (range 1 to 89%). Twenty-five studies provided data on injury severity scores (ISS) of participants. The mean ISS for studies reporting ISS was 24, range 15 to 33. The inclusion criteria for participants varied. Three studies used a systolic blood pressure (SBP) below 80 mmHg [38,37,46], 15 RCTs used 90 mmHg [29,31,33,34,39-41,43,44,48,51,52,56,61,63] and 3 studies used 100 mmHg [30,42,53]. Only one RCT used base deficit as an inclusion criterion [61]. Seventeen studies provided data on the percentage of participants receiving blood transfusions (overall mean 74%, range: 5 to 100%) [25,26,31,33,35,36,38,39,42,46,55-57,61,62]. Enrolled patients receiving massive transfusion (over 10 units of RBC in 24 hours) varied from 6 to 100% (mean 30%) [25,36,37,42,53,57,61].

**Figure 2:**
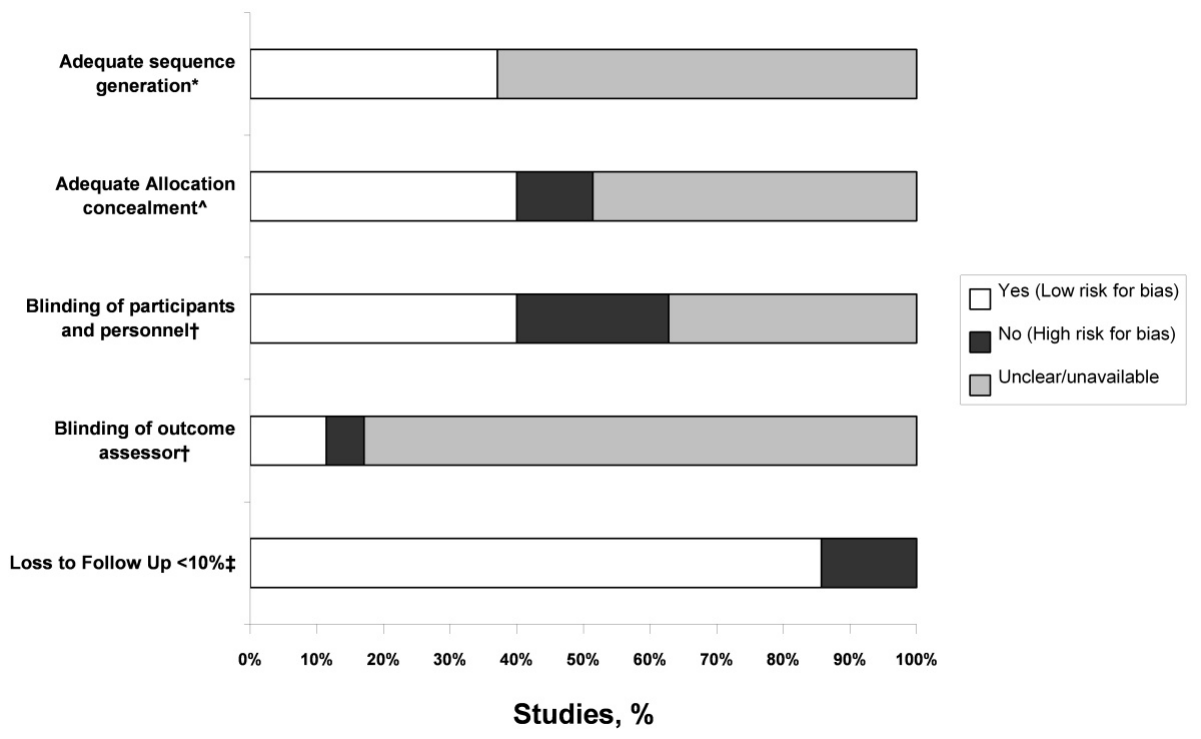
**Risks for bias in included RCTs**. We assessed study risk for bias according to recommendations from the Cochrane Collaboration [23]. *Whether the study reported methods of randomization sufficiently to meet current CONSORT guidelines for true random allocation of participants [71]. ^ Whether the study reported methods to conceal allocation sufficiently to determine whether the chosen intervention for a participant could have been predicted in advance. † Whether the study reported methods by which patients, staff or assessors were prevented from knowing the intervention given to each participant. ‡ Whether the study described loss-to-follow up figures.

Methodological quality is summarized in Additional file 3 and Figure 2[71]. Only 12 studies described adequate sequence generation methods. Allocation concealment was detailed in 23 studies and adequate in 13. Twenty-one trials did not report blinding, 14 reported blinding of either participants or personnel and 4 of these also reported blinding of the outcome assessor. Most studies (*n *= 26) had no loss of patients, and five had less than 10% loss to follow-up. Only one study used good methodological practices in all areas examined [56]. There was no trend to improvement in methodological quality over time.

### Blood and blood saving strategies (seven trials enrolling 1,374 participants)

Seven RCTs were identified which examined blood products (*n *= 2) or blood saving strategies (*n *= 5). Of the two RCTs that looked at blood product administration, one compared platelet therapy with fresh frozen plasma (FFP) for the prevention of microvascular bleeding [25]. The second compared leucodepleted versus standard blood products in terms of infection [26], micro-chimerism [27], and acute lung injury [28]. Five RCTs looked at methods of reducing allogeneic blood use. One assessed red blood cell (RBC) salvage in abdominal injury [29] and four trials evaluated a blood substitute, (PolyHeme, Northfield Laboratories Inc., Evanston, Illinois, USA [30,31] or diaspirin cross-linked hemoglobin - DCLHb, Baxter Healthcare, Round Lake, Illinois, USA [32,33]).

Mortality rates were not affected by platelet administration [25], leucodepleted blood products [26], or cell salvage [29]. Only two of the four blood substitute RCTs reported mortality and neither identified a difference in outcome [31,33]. Three of the blood substitute studies reported morbidity outcomes (MOF, ARDS or infection) with no significant findings [31-33].

Transfusion requirements were reduced by cell salvage at 24 hours [29]. Three of the blood substitute studies also reported a significant reduction in RBC requirements [30,31,33]. The fourth study of DCLHb did not report transfusion use [32]. There was no difference in microvascular bleeding in the RCT comparing platelet and FFP transfusions [25].

Four trials reported coagulation outcomes [25,29,31,32]. Neither platelet transfusion, when compared to FFP [25], nor cell salvage [29] led to any significant improvement in coagulation. DCLHb did not affect activated partial thromboplastin time (APTT) [32], but patients receiving PolyHeme had significantly increased rates of prolonged prothrombin time (PT) and APTT, although an imbalance in these parameters was seen at the time of randomization [31].

### Mechanical and surgical management. (two trials enrolling 257 participants)

Only two RCTs were identified. One study examined the use of Pneumatic Anti-Shock Garments (PASG) for traumatic injury [34] and a second investigated whether vascular control of renal vessels during surgery for kidney injury altered outcome [35].

There was a trend to increased mortality in those patients treated with PASG [34]. Transfusion requirements were not altered by either intervention [34,35] and intra-operative blood loss was similarly unaffected during surgery for renal trauma [35]. Neither study reported coagulation results.

### Use of intravenous fluids for resuscitation (18 trials enrolling 3,394 participants)

Twelve of 18 studies compared different resuscitation fluids: colloid vs. colloid (*n *= 1) [36]; colloid vs. crystalloid (*n *= 4) [37-40]; or crystalloid vs. hypertonic saline+/-dextran (HSD) (*n *= 7) [41-47]. The remaining six studies examined fluid administration strategies, including immediate vs. delayed (two RCTs) [48,49]; continuous arteriovenous rewarming (CAVR) (one RCT) [50]; and achievement of hemodynamic goals (three RCTs) [51-53]. The hemodynamic endpoint RCTs evaluated various interventions; the achievement of a certain systolic blood pressure (SBP) using a rapid infusion system [51]; a high or low SBP endpoint [52]; and the effect of increased hemodynamic monitoring against standard care [53].

Mortality was reduced at 24 hours and 30 days with HSD [46], but this was not reproduced in the six other HSD studies [41-45,47]. Delayed fluid administration led to a significant improvement in survival to hospital discharge in one of two studies on timing of fluid therapy [48]. The second study did not find any mortality difference [49]. No RCT of hemodynamic endpoints identified any significant mortality differences [51-53]. CAVR led to a significant reduction in mortality at 24 hours but no difference at hospital discharge [50].

Seven of 18 trials reported other clinical outcomes. Five evaluated the development of ARDS [37,48-51]. A significant increase was reported following albumin administration [37] and a trend was seen with CAVR [50]. Two studies reported MOF, both showing no difference between study arms [36,53]. Five RCTs reported infection data [36,48-50,53] but only Plasma Protein Fraction (PPF) infusion showed a significant difference [36].

There was no difference in transfusion requirements in 10 of the 12 RCTs examining type of fluid administered [36-39,41-44,46,47]. A significant reduction in RBC use was reported at one hour with pentastarch [40] and throughout resuscitation with hypertonic solutions [45]. Transfusion requirements were not affected by timing of fluids [48,49]. Of the three RCTs examining hemodynamic endpoints only the rapid infuser showed a significant reduction in RBC transfusion and only in the first hour [51] CAVR did not affect blood product use [50].

Clotting parameters were reported in seven of these RCTs [36,39,43,44,48,50,51]. Three studies showed a difference: a higher APTT was seen on days 1 to 2 in patients receiving Hetastarch (HES) compared to PPF, but no difference in PT [36]; APTT was improved at 5 to 10 hours in patients receiving fluids via a rapid infusion system [51]; and there was a significantly prolonged PT and APTT in patients receiving immediate compared to delayed fluid therapy, but no significant difference after operative intervention [48].

### Pharmaceutical agents (eight trials enrolling 21,689 participants)

Three of eight pharmaceutical trials reported effects of antifibrinolytics in trauma [54-56]. Aprotinin was compared to heparin [54] and to placebo [55] and tranexamic acid was compared to placebo [56]. Two RCTs (published as one paper [57]) reported the effects of recombinant factor VIIa (rFVIIa) in blunt and penetrating injury. Three *post-hoc *subgroup analyses [58-60] were published from these original data. A phase III RCT examining the efficacy of rFVIIa in the management of traumatic hemorrhage has been recently published [61]. Two RCTs looked at novel drugs examining the effects of a bactericidal protein (rBPI21) [62] and a monoclonal antibody (*rhu*MAb CD18) [63].

All pharmaceutical trials reported a mortality outcome. There was a significant reduction in death due to bleeding and all cause mortality in trauma patients receiving tranexamic acid [56]. The two small aprotinin RCTs did not identify a mortality benefit [54,55]. rFVIIa administration did not affect mortality [57,58,61]. A trend towards reduced mortality was reported at day 15 following administration of rBPI21 [62].

Five trials reported other clinical outcomes. Results from the phase II rFVIIa study reported no difference in MOF rates for blunt injury [57], and a trend to reduction of MOF in the penetrating [57], and the coagulopathic subgroups [58]. For those patients surviving more than 48 hours, there was a significant reduction in MOF rates in blunt trauma [59]. The phase III rFVIIa study reported a trend to reduction of MOF for blunt injury [61]. ARDS rates were significantly reduced in the intervention arms in three RCTs; rFVIIa in blunt injury [57] and the coagulopathic subgroup [58], aprotinin in pulmonary insufficiency [55] and *rhu*MAb CD18 [63]. A trend to reduction of ARDS was reported in the recent rFVIIa RCT in blunt injury [61]. Rates of sepsis were unaffected by rFVIIa in either injury group in this same study [61].

Transfusion outcomes were reported in one of the three RCTs of antifibrinolytic agents [56]. Transfusion use was not altered over a 28-day period following administration of tranexamic acid. In contrast, rFVIIa led to a significant reduction in RBC [57,61] and FFP [61] requirements in blunt injury and a trend to reduction of RBC [57] or total allogeneic transfusion [61] use in penetrating injury. In the coagulopathic subgroup a significant reduction in RBC and FFP use and a trend to a reduction in platelet use was reported at 48 hours [58]. Patients treated with rFVIIa and placebo received significantly greater numbers of massive transfusions if their post-study drug PT remained elevated at one hour [60]. Neither RCT examining novel drugs showed a difference in transfusion requirements [62,63].

Little coagulation data were presented from the antifibrinolytic studies, and none from the novel drug RCTs. In the study where heparin was compared to aprotinin the heparin group was reported to have higher factor assay levels up to day 7 [54]. The RCTs examining rFVIIa in trauma originally did not report coagulation data [57]. In a subsequent report, rFVIIa reduced the mean PT and antithrombin and fibrinogen levels were significantly lower in patients with PT values > 18s [60]. The phase III rFVIIa study reported no difference in disseminated intravascular coagulation (DIC) rates between rFVIIa and placebo [61].

## Discussion

The 35 RCTs identified might be expected to provide a strong evidence base for a single clinical condition. However, the multifactorial nature of trauma hemorrhage, the multiplicity of interventions, issues with trial design, difficulties with the conduct of trauma trials and lack of a coordinated approach mean that only limited conclusions can be drawn. The largest sub group of included RCTs evaluated different strategies for using fluids during resuscitation, but did not consistently identify improvements in outcomes. The RCTs evaluating hemoglobin substitutes reported a reduction in RBC requirements but safety remains a concern [20]. Very few studies were identified evaluating the clinical effectiveness of RBC or blood component therapy. Only two studies were identified which evaluated surgical or mechanical interventions, which is surprising given the interest in damage control surgery [72] and angio-embolization [73]. Tranexamic acid was the only pharmaceutical agent that improved mortality [56].

Two studies reported bleeding endpoints using time taken to achieve hemorrhage control as their endpoint [37,52], all other studies reported surrogate outcomes. Transfusion requirement was commonly used as a surrogate outcome for bleeding, but its use introduces issues with variations in transfusion practice, differences in product type and availability, and survivor bias [74]. Although transfusion for trauma hemorrhage is usually completed within a few hours of injury [75], a large proportion of the transfusion data was reported over a much longer timeframe. Differentiation between early and late transfusion use is an important distinction in understanding the effects of interventions for acute bleeding.

There was no demonstrable association between survival and transfusion requirements, despite evidence from observational studies [76,77]. None of the nine trials reporting a reduction in RBC use had an associated survival improvement [29-31,33,39,45,51,57,61]. Conversely, other studies reported survival benefits but did not observe differences in transfusion use [46,48,56]. No study used correction of coagulopathy as a defined endpoint. Newer methods of assessing hemostasis such as thromboelastography were not used and a definition of coagulopathy was variable and provided by a limited number of trials [32,58,60].

Many of the included trials were poorly designed or conducted, underpowered or recruited small numbers of participants. Recruitment to trauma RCTs can be difficult, not least because of the challenges of enrolling incapacitated patients where informed consent is impossible, although some countries now have recognized processes for emergency consenting. Low patient numbers affect study power and increase the risk of bias, since baseline imbalances between patient groups is likely to occur even if randomization has been rigorous [78]. Only five studies were powered to provide mortality results, and it is likely that the improvement in mortality suggested by the sample size calculations (ranging between 6 and 35%) was over optimistic in many studies [79]. In contrast the CRASH-2 study tested the hypothesis that tranexamic acid would provide a 2% survival benefit which projected a sample size of 20,000 participants [56].

There are limitations to this review. A quantitative analysis was not possible because of the heterogeneity between studies. For example, the inclusion criteria for patients varied widely, such as SBP values for shock. This increases the risk of missing low levels of benefit or harm, which were not large enough to be statistically relevant in any single RCT. The heterogeneity also highlights the importance of working towards uniformity in clinical trials. Attempts were made to identify all relevant RCTs including those in the non-English literature, but some studies may have been missed. Our literature search spanned 60 years, a time frame which has seen trauma care alter significantly. The included RCTs are all from civilian settings, and, therefore, RCT data do not exist to evaluate changes in military practice, although the recent changes in transfusion support for trauma patients have been driven by military data. There were no eligible RCTs examining, for example, the role of tourniquets and, therefore, this area has not been addressed in our review, although RCTs may not be indicated for every intervention.

## Conclusions

The acute management of trauma hemorrhage has been evaluated in a large number of trials but these have not in the main produced results that have changed management or improved outcomes. This systematic review set out to examine RCTs, as the most robust form of study design and in so doing observational data have not been identified and appraised. However, it demonstrates that the difficulties associated with recruitment, design and conduct of trauma trials can be overcome to produce better quality RCTs. As our understanding of the pathophysiology of trauma hemorrhage grows, a coordinated strategy is required for this globally important condition.

## Key messages

• A total of 35 RCTs were identified relating to the management of trauma haemorrhage, but due the multifactorial nature of hemorrhage, the multiplicity of the RCT interventions, issues with trial design and difficulties with the conduct of trauma trials, only limited conclusions could be drawn.

• The RCT literature did not demonstrate a correlation between reduction of transfusion requirement and improvement in the survival of their participants, even though the observational literature has reported such an association.

• Large, well-conducted studies with pragmatic endpoints are required to improve our understanding of the complex interplay between bleeding and coagulopathy, transfusion requirements and mortality.

• The CRASH-2 study has confirmed that large, well-conducted trauma studies are achievable.

## Abbreviations

APTT: activated partial thromboplastin time; ARDS: acute respiratory distress syndrome; CAVR: continuous arteriovenous rewarming; DCLHb: diaspirin cross linked hemoglobin; DIC: disseminated intravascular coagulation; FFP: fresh frozen plasma; HES: Hetastarch; HSD: hypertonic saline dextran; ICTRP: International Clinical Trials Registry Platform; ISS: injury severity score; MOF: multi organ failure; NHSBT SRI: National Health Service Blood and Transplant Systematic Review Initiative; PASG: pneumatic anti-shock garment; PPF: plasma protein fraction; PT: prothrombin time; RBC: red blood cell; rBPI21: bactericidal/permeability-increasing protein; RCT: randomized controlled trial; rFVIIa: recombinant activated factor VII; *rhu*MAbCD18: recombinant humanized monoclonal antibody against CD18; SBP: systolic blood pressure.

## Competing interests

The authors declare that they have no competing interests.

## Authors' contributions

NC contributed to study design, acquisition of data, analysis and interpretation of data, drafted and revised the article. SH contributed to analysis and interpretation of data, and revision of the article. CD contributed to study design, acquisition of data, and revision of the article. CH and KB contributed to study conception and design, and revision of the article. SS contributed to study conception and design, acquisition of data, analysis and interpretation of data, and revision of the article.

## Supplementary Material

Additional file 1 **Search strategy**. This file contains full documentation of the comprehensive search strategy completed for this systematic review. Click here for file

Additional file 2 **Included randomized controlled trials**. This file contains a table listing all the included RCTs within this systematic review, including groups of patients examined, intervention and comparator arms and main clinical outcomes of each study. Click here for file

Additional file 3 **Quality assessment of included published randomized controlled trials**. This file includes a table detailing the quality assessment of all included RCTs in this systematic review. It particularly focusses on sequence generation, allocation concealment, blinding and incomplete outcome data. Click here for file
