# Supplementary material for: The acute management of trauma hemorrhage: a systematic review of randomized controlled trials
Source: Crit Care. 2011 Mar 9;15(2):R92. doi: 10.1186/cc10096 (PMC3219356; doi:10.1186/cc10096)
Supplement: Additional file 1 — Search strategy. This file contains full documentation of the comprehensive search strategy completed for this systematic review. [file cc10096-S1.DOC]

**Additional File 1: Search Strategies**

**THE COCHRANE LIBRARY (CENTRAL)**

#1 MeSH descriptor Wounds and Injuries explode all trees

#2 MeSH descriptor Critical Care explode all trees

#3 MeSH descriptor Accidents explode all trees

#4 MeSH descriptor Shock explode all trees

#5 MeSH descriptor Trauma Severity Indices explode all trees

#6 MeSH descriptor Trauma Centers explode all trees

#7 MeSH descriptor Emergency Treatment explode all trees

#8 MeSH descriptor Resuscitation explode all trees

#9 MeSH descriptor Traumatology, this term only

#10 MeSH descriptor Military Medicine explode all trees

#11 (trauma* OR polytrauma* OR (pelvi* NEAR/2 fracture*) OR (femoral NEAR/2 fracture*) OR (femur NEAR/2 fracture*))

#12 (accident* OR resuscitat* OR wounded OR rupture* OR lacerat* OR crush* OR septic shock OR shocked OR stabb* OR shoot* OR gunshot* OR bomb* OR emergenc* OR disaster* OR casualt* OR catastroph* OR combat* OR battlefield*):ti

#13 (coagulopath* OR (abnormal* NEAR/2 coagulation) OR hyperfibrinolysis):ti

#14 (injur* OR accident* OR wound* OR rupture* OR lacerat* OR crush* OR shock OR shocked OR stabb* OR shoot* OR gunshot*) NEAR/5 (critical* OR massive* OR serious* OR severe* OR life-threatening*)

#15 ((uncontroll* OR ongoing OR massive* OR critical* OR serious* OR severe* OR life-threatening* OR pelvic OR pelvis OR abdominal* OR arterial* OR coagulopath* OR catastrophic* OR control* OR management) AND (haemorrhag* OR hemorrhag* OR bleed* OR bloodloss* OR blood loss*)):ti

#16 haemorrhag* shock* OR hemorrhag* shock* OR hypotensive resuscitation OR hypovolemic shock OR hypovolaemic shock

#17 (exsanguin* OR hypovolemi* OR hypovolaemi*):ti

#18 #1 OR #2 OR #3 OR #4 OR #5 OR #6 OR #7 OR #8 OR #9 OR #10 OR #11 OR #12 OR #13 OR #14 OR #15 OR #16 OR #17

**MEDLINE (Ovid)**

1. "Wounds AND Injuries"/ OR exp Abdominal Injuries/ OR Amputation, Traumatic/ OR Blast Injuries/ OR exp Craniocerebral Trauma/ OR exp Fractures, Bone/ OR exp Fractures, Cartilage/ OR exp Hip Injuries/ OR Lacerations/ OR Leg Injuries/ OR Femoral Fractures/ OR exp Multiple Trauma/ OR Neck Injuries/ OR exp Rupture/ OR exp Shock, Traumatic/ OR exp Crush Syndrome/ OR exp Soft Tissue Injuries/ OR exp Spinal Cord Injuries/ OR exp Spinal Injuries/ OR exp Thoracic Injuries/ OR exp Trauma, Nervous System/ OR exp Wounds, Penetrating/

2. exp Critical Care/

3. exp Accidents/

4. exp Shock/

5. exp Trauma Severity Indices/

6. Traumatology/

7. Trauma Centers/

8. Emergency Treatment/

9. Resuscitation/

10. Military Medicine/

11. (trauma* OR polytrauma* OR (pelvi* adj2 fracture*) OR (femoral adj2 fracture*) OR (femur adj2 fracture*)).tw.

12. (accident* OR resuscitat* OR wounded OR rupture* OR lacerat* OR crush* OR septic shock OR shocked OR stabb* OR shoot* OR gunshot* OR bomb* OR emergenc* OR disaster* OR casualt* OR catastroph* OR combat* OR battlefield*).ti.

13. (coagulopath* OR (abnormal* adj2 coagulation) OR hyperfibrinolysis).ti.

14. ((injur* OR accident* OR wound* OR rupture* OR lacerat* OR crush* OR shock OR shocked OR stabb* OR shoot* OR gunshot*) adj5 (critical* OR massive* OR serious* OR severe* OR life-threatening*)).tw.

15. ((uncontroll* OR ongoing OR massive* OR critical* OR serious* OR severe* OR life-threatening* OR pelvic OR pelvis OR abdominal* OR arterial* OR coagulopath* OR catastrophic* OR control* OR management) AND (haemorrhag* OR hemorrhag* OR bleed* OR bloodloss* OR blood loss*)).ti.

16. (haemorrhag* shock* OR hemorrhag* shock* OR hypotensive resuscitation OR hypovolemic shock OR hypovolaemic shock).tw.

17. (exsanguin* OR hypovolemi* OR hypovolaemi*).ti.

18. or/1-17

**EMBASE (Ovid)**

1. (trauma* OR polytrauma* OR (pelvi* adj2 fracture*) OR (femoral adj2 fracture*) OR (femur adj2 fracture*)).mp.

2. (coagulopath* OR (abnormal* adj2 coagulation) OR hyperfibrinolysis).ti.

3. ((injur* OR accident* OR wound* OR rupture* OR lacerat* OR crush* OR shock OR shocked OR stabb* OR shoot* OR gunshot*) adj5 (critical* OR massive* OR serious* OR severe* OR life-threatening*)).ab.

4. ((uncontroll* OR ongoing OR massive* OR critical* OR serious* OR severe* OR life-threatening* OR trauma* OR pelvic OR pelvis OR abdominal* OR arterial* OR coagulopath* OR catastrophic* OR control* OR management) AND (haemorrhag* OR hemorrhag* OR bleed* OR bloodloss* OR blood loss*)).ti.

5. (haemorrhag* shock* OR hemorrhag* shock* OR hypotensive resuscitation OR hypovolemic shock OR hypovolaemic shock).mp.

6. (exsanguin* OR hypovolemi* OR hypovolaemi*).ti.

7. ((massive* OR critical* OR serious* OR severe* OR life-threatening*) adj3 (injur* OR accident* OR wound*)).ti.

8. or/1-7

CURRENT CONTROLLED TRIALS
trauma% AND (transfus% OR resuscitat% OR FFP OR antifibrinoly% OR thromboelastog% OR thrombelastog%)

OR

“severe trauma” OR “serious% injur%” OR “serious trauma” OR polytrauma

OR

“critical injury” OR “critically injured” OR “life-threatening injury” OR “seriously wounded” OR “haemorrhagic! shock” OR “hypotensive resuscitation” OR “hypovolemic! shock”

OR

“pelvic fracture*” OR “femoral fracture*” OR “femur fracture” OR “fractured pelvis” OR “fractured femur”

**CLINICALTRIALS.GOV**

1. TRAUMA* OR MASSIVE* OR RESUSCITAT* OR HYPOVOL* OR COAGULOPATH* OR THROMBOELASTO* OR THROMBELASTO*

2. (TRAUMA OR RESUSCITATION OR HYPOVOLEMIC OR HYPOVOLEMIA OR COAGULOPATHY OR THROMBOELASTOGRAPHY OR THROMBELASTOGRAPHY) AND RANDOMIZED AND TRAUMA

3. 1 OR 2

**WHO ICTRP**

TRAUMA* OR MASSIVE* TRANSFUSION* OR RESUSCITAT* OR HYPOVOL* OR COAGULOPATH* OR THROMBOELASTOGRAPH* OR THROMBELASTOGRAPH* OR (INJUR* NOT BRAIN)
