# Supplementary material for: The acute management of trauma hemorrhage: a systematic review of randomized controlled trials
Source: Crit Care. 2011 Mar 9;15(2):R92. doi: 10.1186/cc10096 (PMC3219356; doi:10.1186/cc10096)
Supplement: Additional file 2 — Included randomized controlled trials. This file contains a table listing all the included RCTs within this systematic review, including groups of patients examined, intervention and comparator arms and main clinical outcomes of each study. [file cc10096-S2.DOC]

**Additional file 2: Included Randomized Controlled Trials**

| **Study** | **Clinical Group of Trauma Patients** | **Intervention details (numbers randomized)** | **Comparator details (numbers randomized)** | **Main (clinical) outcomes** |  | | | |
| --- | --- | --- | --- | --- | --- | --- | --- | --- |
| **Transfusion and alternative strategies** | | | | |  | | | |
| Platelet therapy | | | | |  | | | |
| Reed, 1985 | Adults, likely to require 12+ U RBC within 12hrs | 6U plts with every 12U whole blood (contains ~ 420ml plasma) (n=22) | 2U FFP with every 12U whole blood (~440ml plasma) (n=19) | Prophylactic platelet therapy does not prevent diffuse microvascular bleeding  No difference in mortality |  | | | |
| Leucodepletion |  |  |  |  |  | | | |
| Nathens, 2006 | Adults, requiring RBC within 24hrs | Leukoreduced allogeneic blood (n=156*) | Non-leukoreduced allogeneic blood (n=168*) | No difference in infection rates (RR = 0.84 (0.55-1.3)). No difference in mortality (RR=1.19 (0.73-1.9)) |  | | | |
| Utter, 2006 *(sub group analysis*) |  | (n=35) | (n=32) | No significant difference in development of transfusion-associated microchimerism (RR = 1.32 (0.65-2.66)) |  | | | |
| Watkins, 2008 *(sub group analysis)* |  | (n=132) | (n=136) | No significant difference in incidence or timing of acute lung injury (RR = 0.97 (0.74-1.29)) |  | | | |
| Cell salvage |  |  |  |  |  | | | |
| Bowley, 2006 | Adults, penetrating abdominal injury, SBP<90mmHg, blood loss | Cell save blood salvage: autologous and allogeneic blood transfusions (n=21) | Allogeneic transfusions  (n=23) | Significant reduction in allogeneic blood use (p=0.008).No difference in mortality (p=NSig) |  | | | |
| Blood substitutes |  |  |  |  |  | | | |
| Gould, 1998 | Adults, SBP <100mmHg due to blood loss | ≤ 6U PolyHeme  (n=21) | Allogeneic blood  (n=23) | Significant reduction in RBC use in first 24 hours |  | | | |
| Moore, 2008 | Adults, SBP≤90mmHg, pre-hospital setting | ≤6U PolyHeme in 12 hours (n=350) | Crystalloid  (n=364) | No difference in mortality rates at d 1 & d 30 Significant reduction of RBC use at 12 & 24hr (p<0.001) |  | | | |
| Przybelski, 1999 | Adults, class 2-4 hemorrhagic, hypovolemic shock | 50ml DCLHb or NSaline (n=53)  100ml DCLHb or NSaline  (n=42)  **3 arm trial** | 200ml DCLHb or NSaline  (n=44) | No significant toxicity with low doses of DCLHb  No difference in mortality |  | | | |
| Kerner, 2003 | Adults, SBP <90mmHg | DCLHb, ≤ 1000ml  (n=58) | Standard hemorrhagic shock treatment  (n=63) | No improvement in organ failure rates or mortality at day 5, or overall survival at day 28 Significant reduction of RBC use at d 1 (p=0.007) |  | | | |
| **Mechanical/Surgical** | | | | |  | | | |
| Mechanical |  |  |  |  |  | | | |
| Bickell, 1987 | >12yrs, penetrating abdominal injury, SBP ≤90mmHg | PASG (pneumatic anti-shock garment)  (n=97) | No PASG  (n=104) | No differences in red cell requirements (p=NSig) or mortality (p=0.097) |  | | | |
| Surgery |  |  |  |  |  | | | |
| Gonzalez, 1999 | Adults, penetrating renal injury | Vascular control of renal pedicle (n=29) | No vascular control (n=27) | No differences in nephrectomy rate, transfusion requirements, or blood loss (p>0.05 for all endpoints) |  | | | |
| **Fluids used for resuscitation** | | | | |  | | | |
| Colloid vs. colloid |  |  |  |  |  | | | |
| Shatney, 1983 | Adults, multisystem injury &/or traumatic shock | Hetastarch - HES  (n=16) | Plasma protein fraction  (n=16) | No differences in fluid or RBC volumes required. No difference in mortality |  | | | |
| Colloid vs. crystalloid | | | | |  | | | |
| Lucas, 1980 | SBP <80mmHg | Human albumin solution (n=46) | No albumin (n=48) | Significant increase in pulmonary complications with albumin use. No difference in RBC use |  | | | |
| Moss, 1981 | Adults, SBP ≤80mmHg, or required ≥5U RBC | Ringer's Lactate (n=20) | Albumin  (n=16) | No difference in pulmonary function  No difference in RBC use |  | | | |
| Nagy, 1993 | Adults, SBP <90mmHg due to hemorrhage | Ringer's Lactate  (n=20) | Pentastarch  (n=21) | No difference in hemodynamic variables, RBC, FFP & platelet use, or mortality  Significantly less PES required to reach hemodynamic goals (p<0.005) |  | | | |
| Younes, 1998 | Adults, SBP<90mmHg | 10% Pentastarch in 250ml aliquots (n=12) | IS 250ml (n=11) | Significant reduction in volumes of fluid (p <0.001) & RBC at 1hr (p=0.015). No difference in hemodynamic variables or mortality at 24 hr |  | | | |
| Hypertonic vs. crystalloid/colloid | | | | |  | | | |
| Maningas, 1989 | Adults, penetrating injury, SBP ≤90mHg, transported by ambulance | 250 ml HSD (hypertonic dextrose saline)  (n=23) | 250ml Plasmalyte A  (n=25) | Pilot study. HSD shown to be safe No difference in mortality. Trend to reduction of RBC use at 4 hr |  | | | |
| Vassar, 1991 | Adults, SBP ≤100mmHg | HSD = 7.5% NS + initially 4.2% dextran 70, then 6% dextran 70 (n=83) | Ringer's Lactate  (n=83) | Trend to improved survival in patients with head injury (OR = 2.1 (1.0-4.5) No difference in mortality or RBC use to 24 hr |  | | | |
| Vassar, 1993a | Adults, ambulance transport, SBP ≤90mmHg | 250ml HS (hypertonic saline) (n=85)  250ml HSD (n=89)  **3 arm trial** | 250ml Normal Saline (n=84) | No difference in mortality or RBC use |  | | | |
| Vassar, 1993b | Adults, SBP <90mmHg, | 250ml HS (n=45)  250ml HSD-6% (n=39)  250ml HSD-12% (n=42)  **4 arm trial** | 250ml Ringer’s Lactate  (n=39) | No significant difference in mortality or RBC use |  | | | |
| Younes, 1992 | Adults, SBP <80mmHg | 250ml HS (n=35)  250ml HSD (n=35)  **3 arm trial** | 250ml IS (isotonic saline) (n=35) | Significant reduction in crystalloids & blood required. No difference in mortality. |  | | | |
| Younes, 1997 | Adults, hemorrhagic shock, requiring blood transfusion | HSD 250ml bolus (n=101) | IS 250ml bolus (n=111) | Significant improvement in survival at 24hrs (p=0.007) & 30d (p=0.02) No difference in RBC use at 24 hr |  | | | |
| Jousi, 2010 | Adults, severe injury, >1000 ml estimated blood loss | HS = 7.5% NS, 300ml (n=17) *(arms stratified by blood gas reading)* | 300ml conventional fluid (crystalloid or colloid) (n=20) | No difference in mortality (p=1.0) or red cell transfusion requirements (p=0.416) |  | | | |
| Timing of fluids | | | | |  |  |  |  |
| Bickell, 1994 | Adults, penetrating torso injury, SBP ≤90 mmHg | Fluids delayed until operation (n=289) | Immediate iv fluids prior to surgery (n=309) | Significant improvement in survival (p=0.04), no difference in intra-operative blood loss (p=0.11) |  | | | |
| Turner, 2000 | Adults attended by paramedic crew | Early iv fluids  (n=207)° | Iv fluids withheld for the first hour of pre-hospital care (n=194)° | No evidence that protocols recommending pre-hospital fluid administration do harm in blunt trauma patients for death at 6 months OR= 1.07 (0.73-1.54) |  | | | |
| Continuous warmed fluids | | |  |  |  | | | |
| Gentilello, 1997 | Adult, admitted to ITU, core temp ≤34.5°C | Continuous arteriovenous rewarming  (n=29) | Standard rewarming (n=28) | Persistent hypothermia increases total fluid requirements & risk of early death (p=0.059) No difference in RBC(p=0.48), FFP (p=0.23),platelet (p=0.32) & cryoprecipitate use (p=0.16) at 24 hr |  | | | |
| Hemodynamic variables | | | | |  | | | |
| Dunham, 1991 | 14-60 yrs, SBP <90 mmHg, pallor | Rapid infusion system of administering iv fluids (n=11) | Conventional fluid administration (n=17) | No significant differences in blood products required at 24 hr (p=0.82) or mortality |  | | | |
| Dutton, 2002 | Adults, SBP <90 mmHg, ongoing hemorrhage | SBP of 70mmHg  (n=55) | SBP of >100mmHg  (n=55) | No difference in survival |  | | | |
| Velmahos, 2000 | Adults, SBP <100mmHg & PR >100bpm due to bleeding, needing emergent surgery | ‘Optimal’ hemodynamic resuscitation – additional monitoring (n=40) | Standard hemodynamic resuscitation (n=35) | No difference in survival (p=1.0) or RBC use (p=0.449) |  | | | |
| **Pharmaceutical agents** | | | | |  | | | |
| Anti-fibrinolytics |  |  |  |  |  | | | |
| Kolbow, 1977 | Polytrauma | 1000E heparin iv then 200E/kg x3 days infusion  (n=20) | 500,000KIU trasylol iv then 200,000KIU iv every 4 hours for 5 days (n=15) | No difference in survival |  | | | |
| Rosengarten, 1979 | Hypovolaemic shock, major fractures pelvis, femur & tibia | Aprotinin (500,000KIU bolus, 300,000KIU hrly x96 hrs) (n=35) | Placebo (n=35) | Significant reduction of severe pulmonary insufficiency No difference in RBC & platelet use to d 5 |  | | | |
| Roberts, 2010 | Adults, SBP <90mmHg or PR > 110bpm, within 8 hrs of injury | 1g i.v. tranexamic acid over 10min then 1g over 8hrs (n=10,096) | Placebo, 0.9% N saline (n=10,115) | Significant reduction of all-cause mortality and death from hemorrhage (RR = 0.91 (0.85-0.97)) No difference in blood product use to d 28 (RR = 0.98 (0.96-1.01)) |  | | | |
| rFVIIa |  |  |  |  |  | | | |
| Boffard, 2005a | Adults, blunt injury, requiring >6U RBC in 4hrs | rFVIIa (400μg/kg over 3 doses) (n=69) | Placebo  (n=74) | Significant reduction in PRBC use in severe blunt trauma (p=0.02). No difference in mortality (p=0.58) |  | | | |
| Boffard, 2005b | Adults, penetrating injury, requiring >6U RBC in 4hrs | (n=70) | (n=64) | Trend to reduction in PRBC use in severe penetrating trauma (p=1.0). No difference in mortality (p=0.69) |  | | | |
| Rizoli, 2006, *(sub group analysis of coagulopathic patients)* | Blunt and penetrating injury combined | (n=60) | (n=76) | Significant reduction in PRBC (p=0.02) & FFP (p=0.04) use in coagulopathic patients & trend to reduction of platelets (p=0.09) |  | | | |
| Boffard, 2009, *(sub group analysis of patients surviving 48 hours)*  McMullin, 2010 *(sub group analysis of patients with 1 hour post-dose PT)* | Adults, blunt & penetrating injury, requiring >6U RBC in 4hrs  Adults, blunt & penetrating injury, requiring >6U RBC in 4hrs | (n=139)  (n=86) | (n=138)  (n=83) | Significant reduction in risk of ARDS (OR = 0.16 (0.02-0.73)), MOF (OR = 0.05 (0.0-0.89))or MOF & ARDS (OR = 0.16 (0.02-0.81)) in blunt injury  Significantly increased mortality (p≤0.001), massive transfusion (p=0.02), and fewer ITU-free days (p=0.002) in rFVIIa arm with post-dose PT ≥18 secs |  | | | |
| Hauser, 2010 | Adults, blunt & penetrating injury, ongoing bleeding after 4U RBC | rFVIIa (400μg/kg over 3 doses)  (n=226 blunt injury)  (n=47 penetrating) | Placebo  (n=255 blunt injury)  (n=45 penetrating) | No difference in mortality  Significant reduction in blood usage in blunt (p=0.04) and penetrating injury (p=0.04) |  | | | |
| Anti-infective/inflammatory agents | | | | |  | | | |
| Demetriades, 1999 | Adults, requiring 2U RBC, acute hemorrhage | 4mg/kg rBPI21 for 2days, continuous infusion  (n=204) | Placebo  (n=203) | Trend to reduction in mortality and serious adverse events upto d15 (HR = 0.79 (0.58-1.07)) No difference in RBC use at 24 hr & d 15 |  | | | |
| Rhee, 2000 | Adults, SBP ≤90, blunt or penetrating injury | rhuMAb CD18:  0.5mg/kg (n=21)  1mg/kg mAb(n=47)  2mg/kg mAb(n=18)  **4 arm trial** | Placebo  (n=30) | No difference in infection rates or RBC & FFP use |  | | | |

* In the study by Nathens, these figures are the numbers of patients eligible for enrolment, numbers randomised were 929 for leucoreduced and 935 for standard blood, but due to many patients not receiving a transfusion, the numbers included were for smaller.

° In the study by Turner, the figures relate to the number of paramedics randomised to each fluid protocol, numbers of patients included are; early fluids: n=699, delayed fluids: n=610

rBPI21 - Bactericidal/Permeability-Increasing Protein; NS - not stated; NSig - non significant; OR - odds ratio; HR - hazard ratio; RR - relative risk (Where HR, OR or RR figures are given, the 95% confidence intervals are provided in brackets); U - units; FFP - fresh frozen plasma; SBP - systolic blood pressure; RBC - red blood cell; DCLHb - diaspirin cross-linked haemoglobin; i.v. - intravenous
