# Supplementary material for: The acute management of trauma hemorrhage: a systematic review of randomized controlled trials
Source: Crit Care. 2011 Mar 9;15(2):R92. doi: 10.1186/cc10096 (PMC3219356; doi:10.1186/cc10096)
Supplement: Additional file 3 — Quality assessment of included published randomized controlled trials. This file includes a table detailing the quality assessment of all included RCTs in this systematic review. It particularly focusses on sequence generation, allocation concealment, blinding and incomplete outcome data. [file cc10096-S3.DOC]

**Additional File 3: Quality Assessment of included published RCTs.**

|  | **Adequate sequence generation?** | **Adequate allocation concealment?** | **Blinding described?** | | | | **Incomplete outcome data addressed?** |
| --- | --- | --- | --- | --- | --- | --- | --- |
|  |  |  | **Participants and personnel** | **Outcome assessor** | | |  |
| **Transfusion and alternative strategies** | | | | | | | |
| Platelet therapy |  |  |  | |  | |  |
| Reed, 1985 | U:‘randomized’ | NR | Y | | NR | | No loss to F/U |
| Leucodepletion |  |  |  | |  | |  |
| Nathens, 2006 | Y:‘permuted block scheme’ | Y: ‘sealed opaque envelopes’ | Y | | NR | | 19%(standard care) vs. 15% (leucodepleted) lost to F/U reasons given per arm |
| Cell salvage |  |  |  | |  | |  |
| Bowley, 2006 | Y:‘computer generated’ | U:‘envelopes’ | N | | NR | | No loss to F/U |
| Blood substitutes |  |  |  | |  | |  |
| Gould, 1998 | U:‘randomized’ | U: ‘sealed envelopes’ | N | | NR | | No loss to F/U |
| Moore, 2008 | U:‘randomized’ | NR | NR | | NR | | No loss to F/U |
| Przybelski, 1999 | Y:‘computer generated’ | Y: ‘sealed coded boxes’ | NR | | NR | | No loss to F/U |
| Kerner, 2003 | Y:‘computer generated’ | Y: ‘pharmacy blinded boxes’ | Y | | N | | 9% (DCLHb) vs. 2% (standard) lost to F/U, reasons given per arm |
| **Mechanical/Surgical** | | | | | | | |
| Mechanical |  |  |  | |  | |  |
| Bickell, 1987 | U:‘randomized’ | N:‘alternate day’ | N | | NR | | No loss to F/U |
| Surgical |  |  |  | |  | |  |
| Gonzalez, 1999 | U:‘randomized’ | Y: ‘sealed opaque envelope’ | N | | NR | | No loss to F/U |
| **Fluids used for resuscitation** | | | | | | | |
| Colloid vs. colloid |  |  |  | |  |  | |
| Shatney, 1983 | U: ‘randomized’ | N: ‘alternating’ | NR | | NR | | No loss to F/U |
| Colloid vs. crystalloid | | | | |  | |  |
| Lucas, 1980 | U: ‘randomized’ | NR | NR | | NR | | No loss to F/U |
| Moss, 1981 | U: ‘randomized’ | U: ‘sealed envelope’ | NR | | NR | | No loss to F/U |
| Nagy, 1993 | U: ‘randomized’ | NR | NR | | NR | | No loss to F/U |
| Younes, 1998 | U:‘randomized’ | U:‘closed envelopes’ | NR | | NR | | No loss to F/U |
| Hypertonic vs. crystalloids/colloid | | | | |  | |  |
| Maningas, 1989 | U: ‘randomized’ | N: ‘alternate day’ | Y | | NR | | No loss to F/U |
| Vassar, 1991 | Y: ‘table of random numbers’ | Y:‘coded identical solutions’ | Y | | NR | | No loss to F/U |
| Vassar, 1993a | Y:‘computer generated’ | Y:‘coded identical solutions’ | Y | | Y | | 12% in total lost to F/U,  reasons given but not per arm |
| Vassar, 1993b | Y:‘computer generated’ | Y: ‘coded identical solutions’ | Y | | Y | | Lost to F/U: LR (31%), HS (14%), HSD-6% (22%), HSD-12% (14%), reasons given per arm |
| Younes, 1992 | U: ‘randomized’ | Y:‘coded identical solutions’ | Y | | NR | | No loss to F/U |
| Younes, 1997 | U: ‘randomized’ | Y: ‘coded identical solutions’ | Y | | Y | | 4% HSD vs. 3% IS lost to F/U, reasons given but not per arm |
| Jousi, 2010 | U: ‘randomized’ | NR | NR | | NR | | No loss to F/U |
| Timing of fluids |  |  |  | |  | |  |
| Bickell, 1994 | U: ‘randomized’ | N: ‘alternate day’ | N | | NR | | No loss to F/U |
| Turner, 2000 | Y:‘computer generated’ | NR | N | | N | | No loss to F/U |
| Continuous warmed fluids | | |  | |  | |  |
| Gentilello, 1997 | Y:‘table of random numbers’ | U:‘concealed’ | N | | NR | | No loss to F/U |
| Hemodynamic variables | | | | | | | |
| Dunham, 1991 | U:‘randomized’ | NR | NR | | NR | | No loss to F/U |
| Dutton, 2002 | U: ‘randomized’ | NR | NR | | NR | | No loss to F/U |
| Velmahos, 2000 | Y:‘computer generated’ | U: ‘sealed envelope’ | N | | NR | | No loss to F/U |
| **Pharmaceutical agents** | | | | | | | |
| Anti-fibrinolytics |  |  |  | |  | |  |
| Kolbow, 1977 | U:‘randomized’ | NR | NR | | NR | | No loss to F/U |
| Rosengarten, 1979 | U:‘randomized’ | Y:‘unlabelled coded ampoules’ | Y | | NR | | No loss to F/U |
| Roberts, 2010 | Y:‘computer generated’ | Y:‘identical treatment packs’ | Y | | Y | | 0.4% (TXA) vs. 0.5% (placebo) lost to F/U, reasons given per arm |
| rFVIIa |  |  |  | |  | |  |
| Boffard, 2005a | U:‘randomized’ | NR | NR | | NR | | 9% lost to F/U –reasons given, but not per arm |
| Boffard, 2005b | U:‘randomized’ | NR | NR | | NR | | 6% lost to F/U –reasons given, but not per arm |
| Hauser, 2010 | Y: ‘random permuted blocks, interactive voice system’ | Y: ‘ patient-specific boxes’ | Y | | NR | | Blunt injury: 1.4% (rFVIIa) vs. 5.1% (placebo) lost to F/U  Penetrating injury: 10.7% (rFVIIa) vs. 15.6% (placebo) lost to F/U, reasons given per arm |
| Anti-infective/inflammatory agents | | | | |  | |  |
| Demetriades, 1999 | U:‘randomized’ | Y: ‘pharmacy’ | Y | | NR | | No loss to F/U |
| Rhee, 2000 | Y:‘interactive voice system’ | Y:‘identical coded solutions’ | Y | | NR | | No loss to F/U |

U - insufficient data to be able to make an assessment; NR - not reported; Y - reported and adequate methodology; N - reported and inadequate methodology; F/U - follow-up; LR - Lactated Ringer’s solution; HS - hypertonic saline; HSD - hypertonic dextran saline; IS - isotonic saline; TXA - tranexamic acid; rFVIIa - recombinant activated factor VII
